# Non-occupational physical activity during pregnancy and the risk of preterm birth: a meta-analysis of observational and interventional studies

**DOI:** 10.1038/srep44842

**Published:** 2017-03-22

**Authors:** Ju Wen, Pengcheng Xun, Cheng Chen, Minghui Quan, Ru Wang, Yu Liu, Ka He

**Affiliations:** 1 Key Laboratory of Exercise and Health Sciences of the Ministry of Education, Shanghai University of Sport, Shanghai, China; 2 Department of Epidemiology and Biostatistics, School of Public Health-Bloomington, Indiana University, Bloomington, IN, USA

## Abstract

A meta-analysis was conducted to evaluate the association between non-occupational physical activity (PA) during pregnancy and the risk of preterm birth (PTB). By searching PubMed and EMBASE from inception to August 20, 2016, 25 observational studies (18 cohorts and 7 case-controls) and 12 interventional studies were identified. Comparing the highest to the lowest category of leisure-time PA during pregnancy, the pooled relative risk (RR) of PTB was 0.83 [95% confidence interval (CI) = 0.74–0.93] for cohort studies and 0.60 (95% CI = 0.43–0.84) for case-control studies. No overall significant association was found between domestic or commuting PA and the risk of PTB. In addition, PA intervention did not indicate significant beneficial effect on the risk of PTB. Evidence from the observational studies suggested that leisure-time, but not domestic or commuting, PA during pregnancy was inversely associated with the risk of PTB. The findings were not supported by small-scale and short-term interventional studies. Further research with objective measurement on leisure-time PA is warranted.

Preterm birth (PTB) is the second most common cause of death after pneumonia^1^ among children under 5 years old, accounting for approximately 35% of annual neonatal deaths worldwide^1^,^2^. Active participation in physical activity (PA) during pregnancy is not only beneficial in maintaining pregnant women’s general health condition^3^,^4^, but also reduces their risk of developing chronic diseases such as gestational diabetes mellitus and preeclampsia^5^,^6^. However, the relation between PA during pregnancy and the risk of PTB still remains unclear.

Many previous studies focused on total PA. For example, two studies^7^,^8^ evaluated PA as a single score of energy expenditure including occupational, leisure-time, and domestic PA and found it was not significantly associated with the risk of PTB. It may not be appropriate to combine all domains of PA into a single score and link it to health endpoints including PTB because different domains of PA may have different health impacts. For instance, one study^9^ found that leisure-time PA was inversely associated with the risk of PTB, but commuting PA appeared to be positively related to the risk.

In addition, systematic reviews have suggested that occupational PA such as trunk bend (>1 hour/day), prolonged standing (>4 hours/day), shifting work and heavy physical work, particularly in late pregnancy, may increase the risk of PTB^10^,^11^,^12^,^13^,^14^. However, the associations of non-occupational PA, including leisure-time, domestic, and commuting PA, with the risk of PTB have not been systematically evaluated.

Therefore, we aimed to quantitatively examine the overall association between maternal non-occupational PA during pregnancy and the risk of PTB.

## Methods

### Search strategy

This meta-analysis was performed based on the criteria of Preferred Reporting Items for Systematic reviews and Meta-analyses predefined protocol (PRISMA)^15^. Literature review was systematically conducted in PubMed and EMBASE through August 20, 2016. We used a set of terms related to the exposure (“recreation”, “exercise”, “activity”, “commuting”, “transportation”, “domestic”, “housework”, “household”, or “caregiving”) and another set of terms related to the outcome (“preterm birth”, “preterm delivery”, “preterm labo(u)r”, “early labo(u)r”, “premature birth” or “prematurity”). In addition, some other terms including “job”, “occupational”, and “work” were used because the associations of interest may be reported as secondary results in those studies focusing on the associations between occupational PA and birth outcomes. Google Scholar and the reference lists of the relevant narrative and systematic reviews were searched for additional citations.

### Eligibility criteria

Studies were considered to be eligible for the meta-analysis of observational studies if they were a cohort (prospective or retrospective) or case-control study, and reported relative risk (RR), hazards ratio (HR) or odds ratio (OR) with the corresponding 95% confidence intervals (CIs) of the risk of PTB in relation to any non-occupational PA during pregnancy, or such information could be derived from the presented results. Non-occupational PA included three domains: the leisure time (recreational and sport activities) domain, the domestic (house and gardening work) domain and the commuting (active transportation) domain. For multiple publications with identical exposure and identical outcome using data from the same study, we selected the one with the larger sample size. Studies were considered to be eligible for the meta-analysis of interventional studies if they reported the preterm birth for both the physical activity intervention group and the control group in apparently healthy pregnant women. Two reviewers (J. W. and P. X.) independently reviewed all the relevant articles. Disagreements were resolved by consensus and discussion with a third reviewer (K. H.).

### Quality assessment

All identified observational studies received quality assessment based on the Newcastle-Ottawa quality assessment scale^16^, which evaluates observational studies from three aspects: the selection of study population (4 criteria with 4 stars), the comparability of study population (1 criterion with 2 stars), and the assessment of exposure (3 criteria with 3 stars) for a cohort study or the ascertainment of outcome (3 criteria with 3 stars) for a case-control study. Each star was assigned 1 point with a total of 9 points. The quality of study was considered high if the sum score was ≥8 points, and moderate if the sum score ranged from 5 to 7 points.

### Data extraction

Two co-authors (J. W. and P. X.) independently reviewed each included study and extracted the relevant information. Discrepancies were resolved by group discussion with a third co-author (K. H.). The following information was extracted: last name of the first author, the year when the paper was published, the country where the study was conducted, study design, study period, the number of participants/cases, participant age, exposure and its assessment method, outcome ascertainment, measures of the associations of interest [i.e., RR, HR, or OR with the corresponding 95% CIs], and the covariates adjusted in the final model.

### Data synthesis and analysis

Since PTB is a relatively rare disease, we ignored the distinction among the association measures (i.e., RR, HR, and OR) and undertook a random-effects meta-analysis to estimate the pooled relative risk (RR) and 95% CIs comparing the highest to the lowest category of PA level. If a study did not present multivariable-adjusted models, the unadjusted data was used. When no effect estimate was given, a crude estimate was calculated directly from a 2 by 2 table based on available information. If the estimates were reported for different trimesters respectively, they were combined first with a random-effects meta-analysis model.

Heterogeneity among studies was examined by using Cochran’s *Q* test and quantified by using the *I*^2^ statistic. To reduce the likelihood of drawing a false negative conclusion (type II error), a *P* value of ≤0.10 is considered as statistically significant for Cochran’s *Q* test. Very low, low, moderate, and high degree of heterogeneity were defined as ≤25%, 26–50%, 51–75% and >75%, respectively. Publication bias was assessed by Egger’s regression asymmetry test. The Duval and Tweedie nonparametric “trim and fill” method was used to estimate the pooled association of interest if publication bias was suggested^17^. Sensitivity analyses were performed to evaluate the robustness of our findings: 1) to remove one study from the pooled analysis each time; and 2) to replace random-effects with fixed-effects models.

All analyses were performed with STATA software (Version 14, STATA Corporation LP, College Station, TX). A two-sided *P* value of ≤0.05 was considered statistically significant if not otherwise specified.

## Results

### Study selection process

**Figure 1:**
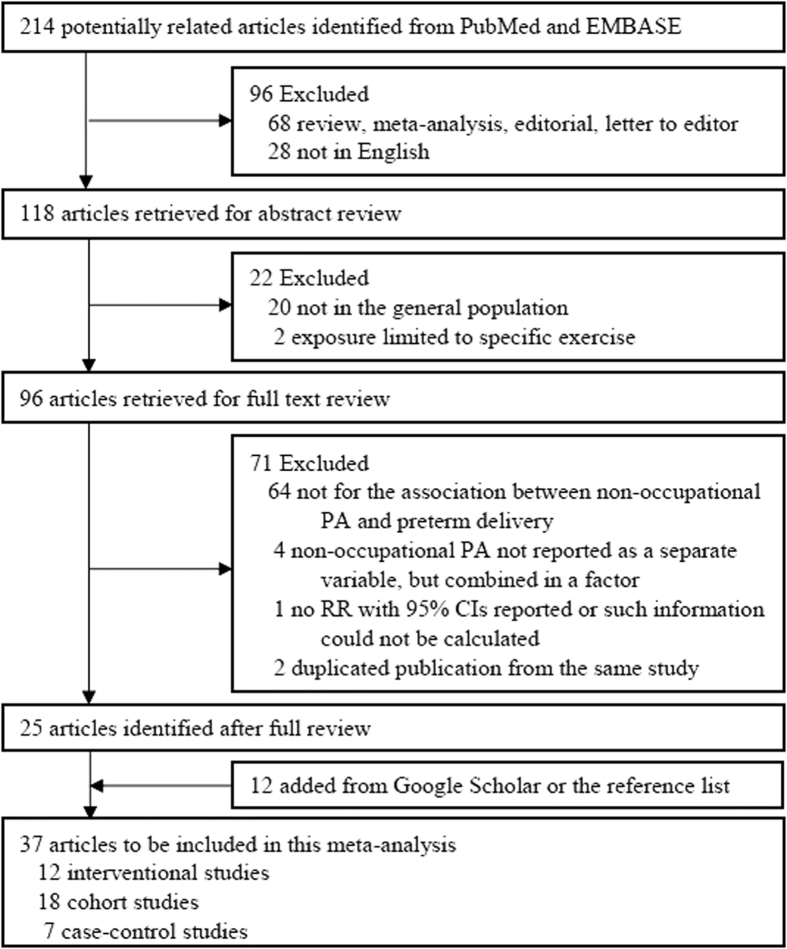
Study selection process.

Figure 1 shows the detailed selection process. We retrieved 214 relevant studies from PubMed and EMBASE. Of them, 189 studies were excluded for one of the following reasons: (1) not an original study; (2) not in English; (3) occupational population, such as nurses, military personnel, physicians; (4) no information on the association of interest and such information cannot be derived from available data; (5) no regular measurement in PA; or (6) duplicated publication. In addition, we identified 12 studies through Google Scholar and the relevant reference lists. Therefore, 37 eligible studies (7 case control studies, 18 cohort studies, and 12 interventional studies) were included in the meta-analysis. All the included 25 observational studies (7 case-control studies and 18 cohort studies) were rated as either high^18^ or moderate^9^,^19^,^20^,^21^,^22^,^23^,^24^,^25^,^26^,^27^,^28^,^29^,^30^,^31^,^32^,^33^,^34^,^35^,^36^,^37^,^38^,^39^,^40^,^41^ quality (see Supplemental Tables S1 and S2).

### Study characteristics

Tables 1 and 2 summarize the characteristics of the included observational studies. Information on leisure-time PA and the risk of PTB were provided in 13 cohort studies^9^,^20^,^25^,^26^,^27^,^28^,^29^,^30^,^32^,^33^,^34^,^35^,^36^ (167,087 participants and 9,096 cases), and 4 case-control studies^21^,^37^,^39^,^41^ (966 cases and 1,685 controls). Of them, 9 studies were conducted in North America^9^,^20^,^21^,^26^,^27^,^34^,^35^,^36^,^41^, 3 in Europe^29^,^30^,^33^, 3 in the Asia-Pacific region^25^,^37^,^39^, and 2 in South America^28^,^32^. Data on domestic PA and the risk of PTB were presented by 7 cohort studies^9^,^22^,^23^,^24^,^25^,^31^,^34^ (11,009 participants and 747 cases), and 3 case-control studies^18^,^21^,^40^ (391 cases and 651 controls). Of these studies, 3 were conducted in North America^9^,^21^,^34^, 2 in Europe^22^,^23^, 2 in the Asia-Pacific region^24^,^25^, 2 in Africa^18^,^31^, and 1 in South America^40^. Information on commuting PA and the risk of PTB was available in 5 cohort studies^9^,^19^,^23^,^24^,^35^ (5,489 participants and 592 cases), and 1 case-control study^38^ (2,230 cases and 3,907 controls). Of these studies, 3 were conducted in Europe^19^,^23^,^38^, 2 in North America^9^,^35^, and 1 in the Asia-Pacific region^24^.

Table 3 summarizes the characteristics of 12 included interventional studies^42^,^43^,^44^,^45^,^46^,^47^,^48^,^49^,^50^,^51^,^52^,^53^ totaling 1,409 pregnant women with 69 PTBs in the PA intervention group, and 1,402 pregnant women with 60 PTBs in the control group. Of these studies, 2 were conducted in the US^42^,^44^, 4 in Sweden^46^,^50^,^51^,^53^, and the rest were in Australia^43^, Brazil^45^,^47^, Iran^52^, Norway^48^, and Spain^49^. The average age of pregnant women ranged from 24.4 to 31.8 years.

### Leisure-time physical activity and the risk of preterm birth

**Figure 2:**
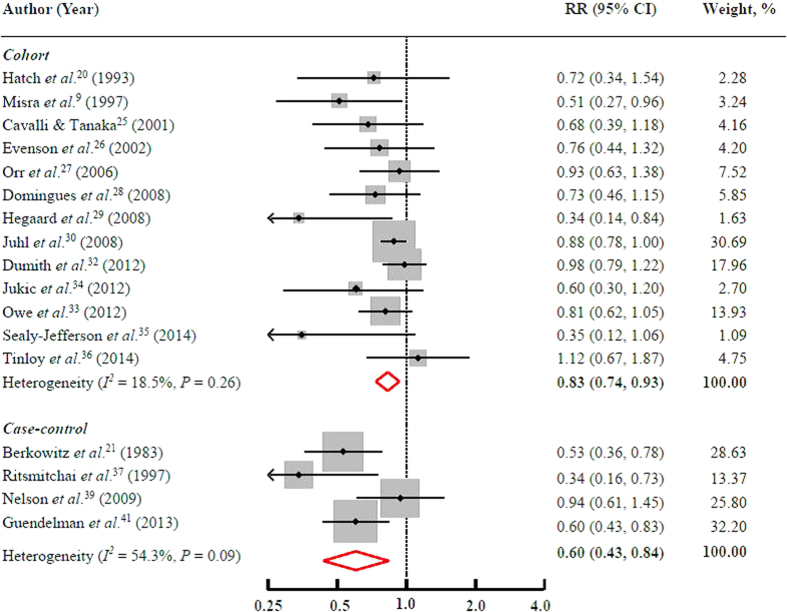
Multivariate adjusted RRs and 95% CIs of the risk of PTB by leisure-time PA levels during pregnancy among 17 observational studies. The pooled estimates (diamond data markers) were obtained using a random-effects model. The dots indicate the RRs for the risk of PTB comparing the highest with the lowest category of leisure-time PA during pregnancy. The size of the shaded square is proportional to the percentage weight of each study. The horizontal lines represent 95% CIs. CI: confidence interval; RR: relative risk.

Thirteen cohort and 4 case-control studies have data on leisure-time PA and the risk of PTB. Comparing the highest to the lowest category of leisure-time PA, the pooled RR of PTB was 0.83 (95% CI = 0.74–0.93) for cohort studies and 0.60 (95% CI = 0.43–0.84) for case-control studies (Fig. 2). No significant heterogeneity (*I*^2^* = *18.5%, *P* = 0.26) was observed in cohort studies, but a moderate heterogeneity was found in case-control studies (*I*^2^* = *54.3%, *P* = 0.09). Since publication bias was detected among cohort studies (Egger’s test: *P = *0.01), we adjusted for the pooled association using the Duval and Tweedie method and the pooled results became 0.78 (95% CI = 0.61–0.997). No evidence of publication bias was found in case-control studies (Egger’s test: *P = *0.63).

Sensitivity analysis indicated that no single study appreciably changed the results, and the pooled associations persisted when a fixed-effects model was used instead of a random-effects one. Notable, the pregnancy period (first, second, third trimester, or mixed) and format (intensity, duration or frequency) of PA assessed were different among these studies. However, the pooled RR was similar [0.83 (95% CI = 0.78–0.88)] when combining data from 11 cohort studies in which leisure-time PA was assessed using frequency (i.e., yes or no, minutes per week, hours per week, times per week and times per month). In addition, the pooled estimate was essentially unchanged [0.80 (0.69–0.94)] when we combined data from 4 cohort studies in which leisure-time PA was measured in the first two trimesters.

### Domestic physical activity and the risk of preterm birth

**Figure 3:**
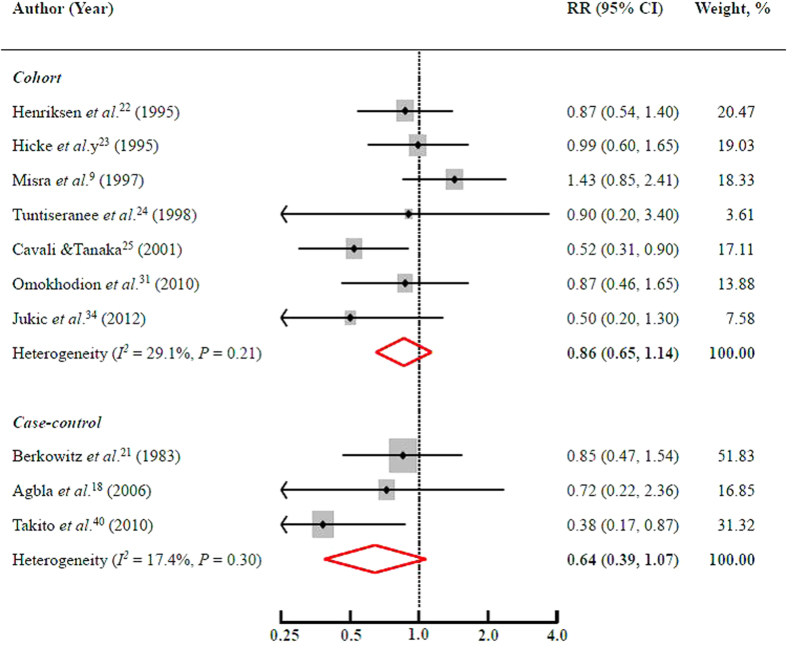
Multivariate adjusted RRs and 95% CIs of the risk of PTB by domestic PA during pregnancy among 10 observational studies. The pooled estimates (diamond data markers) were obtained using a random-effects model. The dots indicate the RRs for the risk of PTB comparing the highest with the lowest domestic PA level during pregnancy. The size of the shaded square is proportional to the percentage weight of each study. The horizontal lines represent 95% CIs. CI: confidence interval; RR: relative risk.

Seven cohort and 3 case-control studies reported results on domestic PA during pregnancy and the risk of PTB. No significant association was revealed. The pooled RR was 0.86 (95% CI = 0.65–1.14) for cohort studies and 0.64 (95% CI = 0.39–1.07) for case-control studies (Fig. 3). Neither significant heterogeneity (*I*^2^ = 29.1% and *P* = 0.21 for cohort; *I*^2^ = 17.4% and *P* = 0.30 for case-control) nor publication bias (Egger’s test: *P* = 0.56 for cohort and *P* = 0.74 for case-control) was evident.

The pooled results generally remained when using a fixed-effects model. However, the pooled association became statistically significant [0.78 (95% CI = 0.60–0.997)] after omitting Misra^9^ *et al*. among cohort studies. Of note, the domestic PA in that study was defined as lifting heavy objects at home. This inverse association was slightly strengthened when further excluding another study^22^ that also included lifting objects at home as the domestic PA [0.74 (95% CI = 0.55–0.998)].

### Commuting physical activity and the risk of preterm birth

Five cohort studies and 1 case-control study presented data on commuting PA during pregnancy and the risk of PTB. No significant association was found among cohort studies comparing the highest to the lowest level of commuting PA (the pooled RR = 1.08; 95% CI = 0.67–1.75). Also, publication bias was not evident (Egger’s test: *P = *0.79). The observed null association was not appreciably altered by any single study and the pooled results persisted when the random-effects model was replaced with a fixed-effects model in the sensitivity analyses.

### Physical activity intervention and the risk of preterm birth

**Figure 4:**
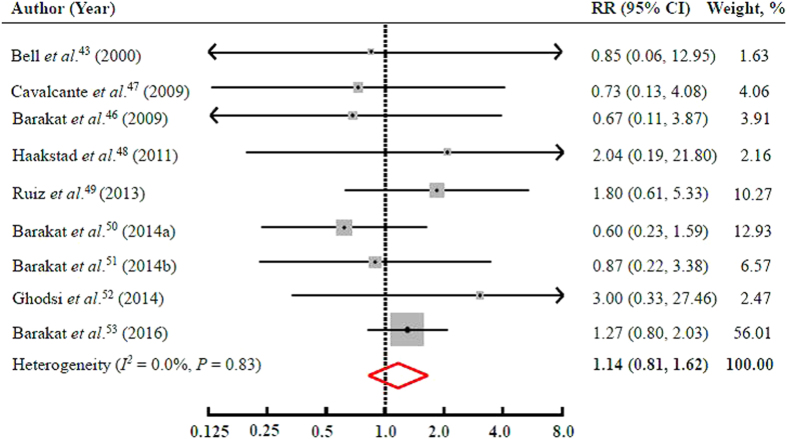
RRs and 95% CIs of the risk of PTB comparing the PA intervention group to the control group among 9 interventional studies. The pooled estimates (diamond data markers) were obtained using a random-effects model. The dots indicate the RRs for the risk of PTB comparing the intervention group to the control group. The size of the shaded square is proportional to the percentage weight of each study. The horizontal lines represent 95% CIs. CI: confidence interval; RR: relative risk.

Twelve interventional studies presented data on PA intervention and preterm birth, and found no significant association (the pooled RR = 1.15; 95% CI = 0.82–1.61). Neither significant heterogeneity (*I*^2^ = 0.0% and *P* = 0.95) nor publication bias (Egger’s test: *P* = 0.78) was documented. Sensitivity analysis indicated that no single study appreciably changed the results, and the pooled associations persisted when a fixed-effects model was used. When two studies specifically on sedentary women and one study on overweight women were excluded, the results were materially unchanged (the pooled RR = 1.14; 95% CI = 0.81–1.62) (Fig. 4).

## Discussion

In the meta-analysis of observational studies, we found a significant inverse association of leisure-time PA during pregnancy with the risk of PTB. Domestic PA was inversely associated with the risk of PTB only if studies defining domestic PA as lifting heavy objects at home were excluded. No significant association was observed between commuting PA and the risk of PTB. However, findings from the observational studies were not supported by interventional studies, which indicate null association.

### Strengths and limitations

To date, this was the largest synthesis of observational studies and interventional studies that quantitatively assessed the association of non-occupational PA during pregnancy with the risk of PTB, which significantly increased the statistical power to detect potential associations. Specifically, we assessed the association separately for each domain of non-occupational PA. Also, all included observational studies were assessed as moderate or high quality using a standardized protocol, so that the likelihood was reduced that the pooled results were substantially biased. Nevertheless, findings from the observational studies should be interpreted in caution because of the following considerations: first, misclassification of PA levels is a concern since PA was assessed with an interview-based questionnaire during pregnancy in the primary studies, which might be subject to recall bias. However, the misclassification is likely to be non-differential and may attenuate the observed associations. To provide more accurate information on PA, objective measurements such as an accelerometer should be used. Second, although the meta-analysis was mainly based on fully adjusted models in the primary studies, the possibility that results were biased by residual confounding or unknown factors could not be completely excluded given the nature of observational study. For example, only a few primary studies considered occupational activity and socioeconomic status in the analysis. This might be an inherent limitation that might affect our findings in the meta-analysis. Third, moderate heterogeneity was observed in a couple of pooled analyses. The sources of heterogeneity include variations in study population, study region, sample size, exposure assessed at different stage of pregnancy, and adjustment for different covariates. We used a random-effects model in concordance with the heterogeneity. Fourth, publication bias due to unpublished data or publications in other languages could not be ruled out. Nevertheless, we used the Duval and Tweedie’s “trim and fill” method to adjust for publication bias. Thus, our findings should not be substantially biased. Fifth, the primary studies did not provide sufficient information to enable us to investigate some important effect modifications such as the age of the women at pregnancy.

By design, intervention studies or clinical trials have certain advantages over the observational studies. However, a few limitations should be acknowledged when interpreting the pooled results from the interventional studies in this meta-analysis. First, the sample size and the number of cases of PTB are relatively small, which indicates the statistical power may not be sufficient. Second, most of the included studies were not designed specifically for studying PTB, i.e., the primary outcome was not PTB (a rare disease) but other outcomes, such as the newborn’s body size^43^,^44^,^46^,^48^, maternal aerobic capacity change^42^,^45^ or weight gain^49^,^51^ during pregnancy, and pregnancy-induced hypertension^53^, which from the other angle explained the low power for the analysis. Third, the most important limitation of the interventional studies was the practical difficulty of maintaining a high compliance in the exercise group due to logistical and family constraints; similarly, the control group may be aware the benefit of exercise and consequently continue or increase their PA, which may explain the null association.

### Comparison with other reviews

Several reviews^54^,^55^,^56^,^57^,^58^,^59^,^60^,^61^,^62^ of observational studies investigated the associations of non-occupational PA during pregnancy with the risk of PTB. While most of them concentrated on leisure-time PA, only three discussed different domains of non-occupational PA during pregnancy in relation to the risk of PTB^55^,^56^,^57^. Of these studies, one systematic review^62^ of literature up to 2014 qualitatively assessed the association of leisure-time PA during pregnancy with the risk of PTB and supported the assertion that healthy pregnant women can engage in low, moderate, and even some vigorous levels of leisure-time PA without risk for preterm birth. Another review^60^ quantitatively combined data from only 4 cohort studies, but found null association between leisure-time PA during pregnancy and the risk of PTB, which may be due to insufficient statistical power.

Several other reviews of interventional studies have discussed the effect of PA during pregnancy on the risk of PTB. For example, a Cochrane review published in 2010^63^, which combined data from 3 studies with a total of 6 PTB cases concluded that the data are insufficient to draw any conclusion. In addition, a meta-analysis of interventional studies^64^, which used maternal weight as the primary outcome, found that PA had a trend of reducing the risk of PTB, though the pooled result from 5 trials (450 participants with 20 cases) was statistically non-significant. One recent systematic review and meta-analysis of 9 interventional studies^65^, including one abstract and one published in other language, concluded that aerobic exercise was not associated with an increased risk of PTB. Similarly, a meta-analysis^62^ of 17 trials found no significant difference in gestational age at delivery between the PA group and the control group.

Although our results are generally consistent with the previous findings, we think the present meta-analysis provides more robust results and additional information to the literature by combining evidence from both observational and interventional studies and focusing on the different domains of non-occupational PA.

### Potential mechanisms

It is generally recognized that pregnant women can get tremendous benefit from regular PA. First, maintaining PA during pregnancy will help pregnant women maintain a general condition of health via improving their lipid profiles and lowering their blood pressures^3^,^4^. Second, regular PA during pregnancy will help women relieve symptoms during pregnancy (e.g., nausea and vomiting)^66^,^67^,^68^ via hormonal and metabolic adaptations associated with improved cardiovascular functioning and alterations in catecholamine release and response^69^. Third, it can help pregnant women reduce the risk of developing chronic diseases such as gestational diabetes mellitus and preeclampsia^5^,^6^ via improved insulin sensitivity, decreased concentrations of proinflammatory cytokines in peripheral circulation, reduced oxidative stress, and improved plasma lipid and lipoprotein concentrations.

There are several explanations for the potential beneficial effect of leisure-time PA on the risk of PTB. First, leisure-time PA may be less strenuous than the other two domains of non-occupational PA. Second, compared with domestic and commuting PA, women who engage in leisure-time PA may represent a select group who are more relaxed, with less stress, since gestational depression is an established risk factor of PTB^70^.

In addition, two cohort studies^9^,^22^ reported results on domestic PA during pregnancy and risk of PTB, in which the domestic PA was defined as lifting objects at home. Non-heavy domestic PA may provide a similar benefit as leisure-time PA after omitting these two studies. Weight lifting may raise the blood pressure and does little or nothing to benefit the heart and cardiovascular system in general, which may explain the change in the result. However, a potential effect of lifting objects at home on risk of PTB cannot be firmly established because the available data were derived from a limited number of studies.

### Implications for clinical practice and future research directions

Based on the best currently available evidence, the results of this meta-analysis show a beneficial effect of leisure-time PA during pregnancy in reducing the risk of PTB. The optimal dose of PA is still unknown, but the present results recommend that appropriate leisure-time PA during pregnancy has the potential to reduce the risk of PTB. This study also indicates that non-heavy domestic PA (e.g., care giving) might benefit the pregnant women with respect to PTB. Future studies, especially well-controlled experimental/interventional studies with sufficient power, are encouraged to better understand the dose-response relationship of leisure-time PA during pregnancy and the risk of PTB.

Our systematic review lends support to the hypothesis that leisure-time physical activity during pregnancy may protect against the incidence of preterm birth. Further studies are needed to identify the most appropriate levels of intensity, duration and frequency of leisure-time PA during pregnancy. Future studies should consider the four domains of PA and potential moderators (e.g., age, race), as well as utilize tools that reliably measure exposure variables. Such studies would provide useful guidelines for pregnant women and clinicians.

In conclusion, evidence from the observational studies suggests that leisure-time PA but not commuting PA during pregnancy was inversely associated with the risk of PTB. Domestic PA may provide a similar benefit, with the exception of lifting heavy objects. Results from the observational studies are not supported by the interventional studies that indicate null associations. Future studies are needed to determine the optimal intensity and frequency of leisure-time PA during pregnancy with respect to the risk of PTB and to elucidate the potential mechanisms.

## Additional Information

**How to cite this article:** Wen, J. *et al*. Non-occupational physical activity during pregnancy and the risk of preterm birth: a meta-analysis of observational and interventional studies. *Sci. Rep.* **7**, 44842; doi: 10.1038/srep44842 (2017).

**Publisher's note:** Springer Nature remains neutral with regard to jurisdictional claims in published maps and institutional affiliations.

## Supplementary Material

Supplementary Information

## Figures and Tables

**Table 1:** Characteristics of 18 cohort studies included in the meta-analysis.

Source	Study period	Age* (years)	No. of cases/participants	Exposure assessment	Exposure Period	Exposure categories	Result	Variables Adjusted	Score of quality assessment
Mamelle *et al*.^19^, France	1977–78	NA	131/1,928	Face to face interview	During the whole pregnancy	Commuting PA: No; Yes.	RR (95% CI): 1.0 (reference); 0.9 (0.6–1.3).	Age, parity, previous poor pregnancy outcome, and pathologic states during pregnancy.	6
Hatch *et al*.^20^, USA	1987–89	27.6 ± 4.5	25/462	Telephone interview or mail questionnaire	During the whole pregnancy	Leisure-time PA: No; Yes.	Crude RR (95% CI)^†^: 1.00 (reference); 0.72 (0.34–1.55).	NA	6
Henriksen *et al*.^22^, Denmark	1989–91	30.0 ± 6.0	152/4,076	Self-administered questionnaire	During the second and third trimester	Domestic PA (lift load kg/day): 0–20; 21–60; 61–100; >100.	Crude RR (95% CI)^†^: 1.00 (reference); 0.71 (0.36–1.38); 0.50 (0.21–1.22); 0.87 (0.54–1.40).	NA	7
Hickey *et al*.^23^, England	1985–88	25.5 ± 5.9	71/592	Self-administered questionnaire	The first 24 to 26 weeks	Domestic PA (intensity): Low; High.	Crude RR (95% CI)^†^: 1.00 (reference); 0.99 (0.60–1.65).	NA	5
						Commuting PA (minute/day): 1–30; 31–60; >60.	Crude RR (95% CI)^†^: 1.00 (reference); 1.14 (0.69–1.87); 1.06 (0.60–1.89).		
Misra *et al*.^9^, USA	1988–89	24.6 ± 6.6	198/1,166	Face to face or telephone interview	During first two trimesters	Leisure-time PA (days): <60; ≥60.	OR (95% CI): 1.00 (reference); 0.51 (0.27–0.95).	Age, race, illicit drug use, prenatal care, height, smoking, insurance/Aid to Families with Dependent Children, parity, obstetrical complication, antepartum hospitalization, and febrile/antibiotic administration.	7
						Domestic PA (Lifting heavy objects): No; Yes.	OR (95% CI): 1.00 (reference); 1.43 (0.85–2.41).		
						Commuting PA (day/week): <4; ≥4.	OR (95% CI): 1.00 (reference); 2.10 (1.38–3.20).		
Tuntiseranee *et al*.^24^, Thailand	1994–95	25.6 ± 5.7	57/1,121	Face to face interview	At the 17^th^ week of gestation (range: 15 to 28 weeks)	Domestic PA (hour/week): Light ≤50; Heavy ≤50; Light >51; Heavy >51.	OR (95% CI): 1.0 (reference); 0.6 (0.2–1.9); 0.7 (0.2–2.6); 0.9 (0.2–3.4).	Age, parity, height, obstetrical complication, baby’s sex, squatting, standing, prenatal care, weight at deliver day, working hours, fast walk, lifting more than 12 kilograms, lifting level, carrying more than 12 kilograms, physical job demand, and work control.	7
						Commuting PA (hour/day): ≤0.5; 0.6–1; ≥1.1.	OR (95% CI): 1.0 (reference); 0.9 (0.3–2.4); 1.4 (0.3–6.1).		
Cavalli &Tanaka^25^, 2001, Japan	1997–98	29.1 ± 5.1	86/1,692	Mail questionnaire	During the whole pregnancy	Leisure-time PA: No; Yes.	OR (95% CI): 1.00 (reference); 0.68 (0.39–1.17).	NA	5
						Domestic PA: Did all of them; Did more than half of them; Did half or less of them.	Crude RR (95% CI)^†^: 1.00 (reference); 0.44 (0.22–0.77); 0.52 (0.31–0.90).		
Evenson *et al*.^26^, USA	1995–98	NA	193/1,699	Telephone interview	During first two trimesters	Leisure-time PA (hour/week): 0; 0.1–2.9 ≥ 3.	OR (95% CI)^‡^: 1.00 (reference); 0.64 (0.34–1.20) 0.76 (0.44–1.32).	Age, smoking, BMI, marital status, education, race, parity, quartiles of energy intake in kilocalorie, and bedrest.	6
Orr *et al*.^27^, USA	1993–95	30.6 ± 6.9	126/922	Face to face interview	During the whole pregnancy	Leisure-time PA: No; Yes.	OR (95% CI): 1.00 (reference); 0.93 (0.63–1.38).	Age, marital status, education, Alcohol consumption, drug use, vaginal bleeding, chronic diseases, previous poor pregnancy outcome, and smoking.	7
Domingues *et al*.^28^, Brazil	2004	26.6 ± 5.0	606/4,147	Face to face interview	During the whole pregnancy	Leisure-time PA (minute): Inactive; Tertile 1; Tertile 2; Tertile 3.	PR (95% CI): 1.00 (reference); 0.73 (0.44–1.22); 0.82 (0.56–1.29); 0.73 (0.46–1.14).	Age, birth interval, family income, race, education, prenatal care, and pregnancy-related morbidities.	8
						Leisure-time PA: No; Yes.	PR (95% CI): 1.00 (reference); 0.55 (0.32–0.96).		
					During first trimester	Leisure-time PA: No; Yes.	PR (95% CI): 1.00 (reference); 0.80 (0.59–1.07).		
					During second trimester	Leisure-time PA: No; Yes.	PR (95% CI): 1.00 (reference); 0.90 (0.66–1.23).		
					During third trimester	Leisure-time PA: No; Yes.	PR (95% CI): 1.00 (reference); 0.50 (0.31–0.80).		
Hegaard *et al*.^29^, Denmark	1989–91	NA	210/5,749	Self-administered questionnaire	During the whole pregnancy	Leisure-time PA (intensity): Sedentary; Light; Moderate to heavy.	OR (95% CI): 1.00 (reference); 0.76 (0.60–1.02); 0.34 (0.14–0.85).	Age, marital status, education, parity, BMI, employment status, psychological distress, and smoking.	7
						Leisure-time PA (hour/week): 0; 1–2; >3.	OR (95% CI): 1.00 (reference); 0.80 (0.53–1.23); 0.70 (0.38–1.28).		
Juhl *et al*.^30^, Denmark	1996–2002	29.4 ± 4.2	4,279/87,232	Computer-assisted telephone interview	During the whole pregnancy	Leisure-time PA (MET*hour/week): 0; >0– ≤5; >5–≤10; >10– ≤15; >15.	HR (95% CI): 1.00 (reference); 0.77 (0.68–0.87); 0.82 (0.74–0.91); 0.83 (0.71–0.96); 0.88 (0.78, 1.00).	Age, gravidity, parity, previous spontaneous abortions, uterine fibroids/malformations/cone biopsy, sub-fecundity, coffee consumption, alcohol consumption, smoking, BMI, job status, working hours, working position, and job strain.	8
						Leisure-time PA: No; Yes.	HR (95% CI): 1.00 (reference); 0.82 (0.76–0.88).		
Omokhodion *et al*.^31^, Nigeria	2008	30.6 ± 6.5	99/997	Face to face interview	During the whole pregnancy	Domestic PA (hour/day): ≤2; >2.	Unadjusted OR (95% CI): 1.00 (reference); 0.87 (0.46–1.65).	NA	7
						Domestic PA (lifting heavy objects): No; Yes.	Unadjusted OR (95% CI): 1.00 (reference); 1.56 (0.91–2.67).		
Dumith *et al*.^32^, Southern Brazil	2007	26.6 ± 6.8	422/2,557	Face to face interview	During the whole pregnancy	Leisure-time PA: No; Yes.	PR (95% CI): 1.00 (reference); 0.98 (0.79–1.22).	Age, marital status, education, family income, parity, prenatal consultation, and twin delivery.	7
Jukic *et al*.^34^, USA	2004–07	29.8 ± 5.0	131/1,552	Telephone interview	First trimester	Leisure-time PA: None; Non-vigorous recreational; ≥1 minute/week of vigorous activity.	OR (95% CI): 1.0 (reference); 1.2 (0.7–2.0); 0.6 (0.3–1.2).	Age, race, education, family income, marital status, alcohol consumption, BMI, smoking, illicit drug use, previous poor pregnancy outcome, vaginal bleeding, vomiting, diabetes, starting to exercise in preparation for getting pregnant, and change in vigorous activity compared to before pregnancy.	7
						Domestic PA: None; Non-vigorous household activity; ≥1 minute/week of vigorous activity.	OR (95% CI): 1.0 (reference); 0.5 (0.3–0.9); 0.5 (0.2–1.3).		
Owe *et al*.^33^, Norway	2000–06	NA	2,667/56,853	Mail questionnaire	First two trimesters	Leisure-time PA: 0; 1–3/month; 1–2/week; 3–5/week; ≥6/week.	OR (95% CI): 1.00 (reference); 0.90 (0.81–1.01); 0.81 (0.73–0.89); 0.76 (0.68–0.86); 0.81 (0.62–1.05).	Age, education, BMI, smoking, and parity.	6
Sealy-Jefferson *et al*.^35^, USA	2001–04	23.0 ± 6.0	135/686	Face to face interview	During the whole pregnancy	Leisure-time PA (intensity): None; Light/moderate; High.	PR (95% CI): 1.00 (reference); 1.01 (0.71–1.44); 0.35 (0.11–1.01).	Age, education, prenatal drug use, smoking, chronic disease history, family resource scales, locus of control, stress, and depression.	8
						Leisure-time PA (minute/week): 0; 1–60; >60.	PR (95% CI): 1.00 (reference); 0.91 (0.61–1.36); 0.77 (0.44–1.36).		
						Commuting PA (minute/day): ≤30; >30.	PR (95% CI): 1.00 (reference); 0.64 (0.43–0.94).		
Tinloy *et al*.^36^, USA	2009–11	27.5 ± 4.6	118/2,370	Telephone interview	During the whole pregnancy	Leisure-time PA (minute/week): <60; 60–149; ≥150.	OR (95% CI): 1.00 (reference); 1.14 (0.71–1.84); 1.12 (0.67–1.86).	Age, race, marital status, education, family income, pre-pregnancy weight category, gestational weight gain, and diabetes or hypertension.	7

Abbreviations: BMI, body mass index; CI, confidence interval; HR, Hazard Ratio; NA, not available; OR, odds ratio; PA, physical activity; PR, prevalence ratio; PTB, preterm birth; RR, relative risk; SD, standard deviation.

*Values are expressed as means ± SDs.

^†^Crude RRs were calculated based on reported percentages of incident PTB, comparing all categories of PA versus the lowest one.

^‡^Overall OR was a pooled OR calculated based on reported ORs of incident PTB in the first two trimesters of pregnancy.

**Table 2:** Characteristics of 7 case-control studies included in the meta-analysis.

Source	Study period	Age* (years)	No. of cases/participants	Method of exposure assessment	Exposure Period	Categories of exposure	Result	Adjusted Variables	Score of quality assessment
Berkowitz *et al*.^21^, USA	1977–78	NA	175/488	Face to face interview	During the whole pregnancy	Leisure-time PA: No; Yes.	OR (95% CI): 1.00 (reference); 0.53 (0.36–0.78).	Race, family income, pre-pregnancy weight, maternal weight gain, infertility history, previous poor pregnancy outcome, vaginal bleeding, and alcohol consumption.	7
						Domestic PA (hour/week): <10; 10–19; 20–29; ≥30.	Crude RR (95% CI)^†^: 1.00 (reference); 0.83 (0.51–1.35); 0.67 (0.40–1.13); 0.85 (0.47–1.55).	NA	
Ritsmitchai *et al*.^37^, Thailand	1993	NA	223/446	Face to face interview	During the whole pregnancy	Leisure-time PA: No; Yes.	OR (95% CI): 1.00 (reference); 0.34 (0.16–0.73).	Pregnancy complications, previous poor pregnancy outcome, physical exertion at work, and prolonged standing at work.	6
Saurel-Cubizolles *et al*.^38^, European countries	1994–97	NA	2,230/6,137	Face to face interview after delivery	During the whole pregnancy	Commuting PA (minute/day): <60; 60–120; >120.	OR (95% CI): 1.00 (reference); 0.94 (0.8–1.1); 0.97 (0.8–1.2).	Age, education, marital status, obstetric history, and country.	6
Agbla *et al*.^18^, 2006, Benin	2000–02	NA	99/203	Face to face interview	During the whole pregnancy	Domestic PA: No; Yes.	Unadjusted OR (95% CI): 1.00 (reference); 0.72 (0.22–2.36).	NA	9
Nelson *et al*.^39^, Thailand	2006–07	26.9 ± 7.0	224/675	Face to face interview	During the whole pregnancy	Leisure-time PA: No; Yes.	OR (95% CI): 1.00 (reference); 0.94 (0.61–1.46).	Age, BMI, and parity.	7
Takito *et al*.^40^, Brazil	2005	27.9 ± 5.2	117/351	Face to face interview	A typical week of the second trimester	Domestic PA (hour/day): <2; 2–3.9; 4–5.9; ≥6.	OR (95% CI): 1.00 (reference); 0.50 (0.23–1.09); 0.56 (0.25–1.27); 0.38 (0.17–0.89).	Age, paid work, education, marital status, high blood pressure, vaginal bleeding, early rupture of membranes, hospitalization and antenatal consultations.	7
Guendelman *et al*.^41^, 2013, USA	2002–03	29.2 ± 5.3	344/1,042	Telephone interview	Second trimester	Leisure-time PA (hour/week of vigorous activity): None; 1–2; ≥3.	Inverse OR (95% CI)^‡^: 1.00 (reference); 0.72 (0.50–1.06); 0.60 (0.43–0.83).	Race and month of delivery.	7

Abbreviations: BMI, body mass index; CI, confidence interval; NA, not available; OR, odds ratio; PA, physical activity; PTB, preterm birth; SD, standard deviation.

*Values are expressed as means ± SDs.

^†^Crude RRs were calculated based on reported percentages of incident PTB, comparing all categories of PA versus the lowest one.

^‡^Inverse OR was calculated based on a set reference leisure-time PA of none, comparing all categories of PA versus the lowest one.

**Table 3:** Interventional studies assessed the effect of physical activity during pregnancy on preterm birth.

Source	Intervention	Duration of intervention, week*	No. of PTBs/participants	Age, year*	Duration of pregnancy, week*	Notes
Collings *et al*.^42^, USA	Aerobic exercise program: 3 times/week	13.4 (7–19)	0/12	26.9 ± 2.8	40.1 ± 1.9	Sedentary women
	Not involved in any regular exercise program	—	0/8	28.0 ± 3.7	39.6 ± 1.9	
Bell, *et al*.^43^, Australia	Continued exercise: ≥5 times/week	NA	1/33	NA	NA	
	Reduced exercise: ≤3 times/week	NA	1/28	NA	NA	
Clapp *et al*.^44^, USA	Weight-bearing exercise: 3–5 times/week	~31.5	1/25	31.0 ± 1.0	39.6 ± 0.3	Sedentary women
	No exercise	~31.6	1/25	31.0 ± 1.0	39.7 ± 0.3	
Santos *et al*.^45^, Brazil	Supervised exercise: 3 times/week, 60 minutes/session (140 ± 88.6 MET-h/week)	17.5 ± 3.3	2/37	26.0 ± 3.4	NA	Overweight women
	Neither encouraged to exercise nor discouraged from exercising (114 ± 62.4 MET-h/week)	18.4 ± 3.9	1/35	28.6 ± 5.9	NA	
Barakat *et al*.^46^, Sweden	Light resistance and toning exercise (3 times/week, 35–40 min/session)	~26.0	2/72	30.4 ± 2.9	39.6 ± 1.1	
	Maintain regular activity during the study period	~26.0	3/70	29.5 ± 3.7	39.7 ± 1.4	
Cavalcante *et al*.^47^, Brazil	Regular, moderate practice of water aerobics for 50 minutes three times a week in an indoor swimming pool with water warmed at 28–30 °C	<20 weeks of pregnancy to delivery	2/34	25.8 ± 4.6	39.2 ± 2.2	
	Would not carry out any regular PA during the entire pregnancy	<20 weeks of pregnancy to delivery	3/37	24.4 ± 5.8	39.1 ± 1.6	
Haakstad *et al*.^48^, Norway	All the women were encouraged to participate in at least 2 out of 3 possible 1-hour dance classes per week, and asked to include 30 minutes of moderate self-imposed PA on the remaining week-days.	17.3 ± 4.1	2/52	31.2 ± 3.7	39.9 ± 1.2	
	The women were neither encouraged to exercise nor discouraged from exercising	18.0 ± 4.3	1/53	30.3 ± 4.4	39.6 ± 1.2	
Ruiz *et al*.^49^, Spain	Standard care plus a structured, supervised, light- to moderate-intensity 50- to 55-minute exercise intervention program 3 days a week	From 9 to 38–39 weeks of pregnancy	9/481	31.4 ± 4	39.6 ± 1.7	
	Standard care: received general nutrition and PA	From 9 to 38–39 weeks of pregnancy	5/481	31.9 ± 4	39.6 ± 1.3	
Barakat *et al*.^50^, Sweden	Exercise group: 85 sessions of general fitness class (3 times/week, 55–60 minutes/session)	From 8–10 to 38–39 weeks of pregnancy	6/138	31.4 ± 3.2	39.7 ± 1.3	
	No exercise	From 8–10 to 38–39 weeks of pregnancy	11/152	31.7 ± 4.5	39.6 ± 1.1	
Barakat *et al*.^51^, Sweden	Supervised physical conditioning program that included three 55- to 60-minute sessions per week	From 9–13 to 39–40 weeks of pregnancy	4/107	31.6 ± 3.9	39.5 ± 1.9	
	No exercise, just received usual information provided by their midwives or health care professionals	From 9–13 to 39–40 weeks of pregnancy	4/93	31.5 ± 3.9	39.2 ± 2.2	
Ghodsi *et al*.^52^, Iran	Exercise continuously on a bicycle ergometer for 15 minutes, 3 times a week; the intensity being 50–60% of maximal heart rate.	From 20–26 weeks of pregnancy to delivery	3/35	23.4 ± 3.7	NA	
	Without any exercise training.		1/35	23.3 ± 3.9	NA	
Barakat *et al*.^53^, Sweden	Exercise group: 3 day/week with 50–55 minutes/session	From 9–11 weeks of pregnancy to the end of the third trimester	37/383	31.6 ± 4.2	39.6 ± 1.7	
	Usual care group: received general advice from their health care providers about the positive effects of PA, were not discouraged from exercising on their own	From 9–11 weeks of pregnancy to the end of the third trimester	29/382	31.8 ± 4.5	39.4 ± 1.9	

Abbreviations: NA, not available; PA, physical activity; PTB, preterm birth; and SD, standard deviation.

*Values are expressed as mean ± SD or mean (minimum-maximum) if not otherwise specified.
