# Supplementary material for: Non-occupational physical activity during pregnancy and the risk of preterm birth: a meta-analysis of observational and interventional studies
Source: Sci Rep. 2017 Mar 22;7:44842. doi: 10.1038/srep44842 (PMC5361095; doi:10.1038/srep44842)
Supplement: Supplementary Information [file srep44842-s1.pdf]

Non-occupational physical activity during pregnancy and the risk of preterm birth: a meta-analysis of observational and interventional studies

Ju Wen, MS[1,2,*], Pengcheng Xun, MD, PhD[2,*], Cheng Chen, MS[2], Minghui Quan, PhD[1], Ru Wang, MD, PhD[1], Yu Liu, MD, PhD[1,†] and Ka He, MD, ScD [2,†]

[1]Key Laboratory of Exercise and Health Sciences of the Ministry of Education, Shanghai University of Sport, Shanghai, China. [2]Department of Epidemiology and Biostatistics, School of Public Health-Bloomington, Indiana University, Bloomington, IN, USA. *These authors contributed equally to this study. Correspondence and requests for materials should be addressed to K. H. (email: kahe@indiana.edu) or Y. L. (email: yuliu@sus.edu.cn)

Table S1 Quality assessment using Newcastle-Ottawa quality assessment scale for cohort studies included in the meta-analysis

Source	Selection*	Comparability†	Outcome‡	Quality
Mamelle *et al.*[19] 1984 France	★★	★★	★★	Moderate
Hatch *et al.*[20] 1993 USA	★★	★★	★★	Moderate
Henriksen *et al.*[22] 1995 Denmark	★★	★★	★★★	Moderate
Hickey *et al.*[23] 1995 England	★	★★	★★	Moderate
Misra *et al.*[9] 1998 USA	★★★	★★	★★	Moderate
Tuntiseranee *et al.*[24] 1998 Thailand	★★	★★	★★★	Moderate
Cavalli &Tanaka[25] 2001 Japan	★	★★	★★	Moderate
Evenson *et al.*[26] 2002 USA	★★	★★	★★	Moderate
Orr *et al.*[27] 2006 USA	★★	★★	★★★	Moderate
Domingues *et al.*[28] 2008 Brazil	★★★	★★	★★★	High
Hegaard *et al.*[29] 2008 Denmark	★★	★★	★★★	Moderate
Juhl *et al.*[30] 2008 Denmark	★★★	★★	★★★	High
Omokhodion *et al.*[31] 2010 Nigeria	★★	★★	★★★	Moderate
Dumith *et al.*[32] 2012 Southern Brazil	★★	★★	★★★	Moderate
Jukic *et al.* [34] 2012 USA	★★	★★	★★★	Moderate
Owe *et al.*[33] 2012 Norway	★	★★	★★★	Moderate
Sealy-Jefferson *et al.*[35] 2014 USA	★★★	★★	★★★	High
Tinloy *et al.*[36] 2014 USA	★★★	★★	★★	Moderate

* Stars awarded for selection. A maximum of 4 stars is to be awarded: 1) the study was population-based (a representative sample of the population under study); 2) the non-exposed cohort drawn from the same community as the exposed cohort; 3) the exposure ascertainment are from structured interview; and 4) the preterm birth was not present at start of study.

† Stars awarded for comparability. A maximum of 2 stars is to be awarded: 1) cohorts are comparable for age; and 2) cohorts are comparable for any additional factor(s).

‡ Stars awarded for outcome. A maximum of 3 stars is to be awarded: 1) assessment of preterm birth is from medical records; 2) the follow-up is long enough for preterm birth to occur; and 3) the follow-up is complete.

Table S2 Quality assessment using Newcastle-Ottawa quality assessment scale for case-control studies included in the meta-analysis

Source	Selection*	Comparability†	Exposure‡	Quality
Berkowitz *et al.*[21] 1983 USA	★★★★	★★	★	Moderate
Ritsmitchai *et al.*[37] 1997 Thailand	★★★	★★	★	Moderate
Saurel-Cubizolles *et al.*[38] 2004 European countries	★★★	★★	★	Moderate
Agbla *et al.*[18] 2006 Benin	★★★★	★★	★★★	High
Nelson *et al.*[39] 2009 Thailand	★★★★	★★	★	Moderate
Takito *et al.*[40] 2010 Brazil	★★★	★★	★★	Moderate
Guendelman *et al.*[41] 2013 USA	★★★	★★	★★	Moderate

* Stars were awarded for selection with a maximum of 4 stars: 1) represents cases with independent validation; 2) cases are consecutive or representative; 3) controls drawn from the same community as the cases; and 4) controls have no preterm birth at the endpoint.

† Stars were awarded for comparability with a maximum of 2 stars: 1) cohorts are comparable for age; and 2) cohorts are comparable for any additional factor(s).

‡ Stars were awarded for exposure with a maximum of 3 stars: 1) the exposure ascertainment are from structured interview; 2) cases and controls have the same method of ascertainment; and 3) Cases and controls have complete follow-up.
